# Transvaginal sonography-guided aspiration of benign ovarian cysts: a retrospective evaluation of safety, feasibility, and economic and environmental implications

**DOI:** 10.1007/s00404-026-08372-9

**Published:** 2026-03-11

**Authors:** H. Endres, M. Cioabla, F. Ebner, W. Janni, I. Juhasz-Boess, L. Jung, S. Lukac, M. Upadhyay, D. Dayan

**Affiliations:** 1 Department of Obstetrics and Gynecology, University Medical Center Freiburg, Hugstetter Str. 55, 79106 Freiburg im Breisgau, Germany; 2 Department of Gynecology and Obstetrics, University Hospital Ulm, Prittwitzstraße 43, 89081 Ulm, Germany; 3 Department of Obstetrics and Gynecology, Alb-Donau Klinikum Ehingen, Spitalstraße 29, 89584 Ehingen (Donau), Germany

**Keywords:** Ovarian cyst, Transvaginal cyst aspiration, Safety, Feasibility, Cost efficiency, Sustainability

## Abstract

**Purpose:**

To evaluate the safety, feasibility, and short-term reliability of transvaginal sonography-guided ovarian cyst aspiration in women with benign-appearing ovarian cysts and to explore its potential economic and environmental implications compared with minimally invasive surgical management.

**Methods:**

This retrospective cohort study included women with sonographically benign ovarian cysts treated with transvaginal sonography-guided aspiration at a tertiary-care hospital between January 2024 and October 2025. Patient characteristics, cyst morphology according to International Ovarian Tumor Analysis (IOTA) Simple Rules, procedural feasibility, complications, and recurrence rates were analyzed. In addition, targeted literature reviews were performed to contextualize reported health care costs and carbon footprints of laparoscopic gynecologic procedures.

**Results:**

Twenty-two women were included. The median age was 58 years (range 31–87), the median cyst diameter was 5.9 cm (range 2.8–10.0), and the median aspirated volume was 55 mL (range 9–600). All cysts fulfilled benign sonographic criteria, most commonly IOTA pattern B1 (77.3%), and were predominantly located in the Douglas space (77.3%). The procedure was technically feasible in 95% of cases and was performed under local anesthesia in all but one patient. No major intra or post-interventional complications occurred. One cyst recurrence (4.5%) was documented during follow-up, noting that most patients were assessed only via self-presentation. Literature data indicate substantially higher health care costs for laparoscopic and robotic gynecologic procedures compared with sonography-guided interventions. Published studies report a mean carbon footprint of approximately 42 kg CO^2^e per laparoscopic procedure, while no data are currently available for sonography-guided cyst aspiration.

**Conclusion:**

Transvaginal sonography-guided ovarian cyst aspiration appears to be a safe and feasible treatment option for carefully selected benign ovarian cysts, with low short-term recurrence and minimal morbidity. Available evidence suggests potential economic advantages and a likely lower environmental impact compared with minimally invasive surgery. Given the small sample size, this approach should be considered a complementary or alternative option in selected patients rather than a first-line treatment and warrants further evaluation in larger prospective studies.

## What does this study adds to the clinical work

**Table:** 

Transvaginal sonography-guided aspiration of benign-appearing ovarian cysts is a safe and feasible minimally invasive alternative with low short-term recurrence. It may reduce costs and environmental impact compared with laparoscopic surgery in selected patients.	

## Introduction

The management of sonographically benign-appearing ovarian cysts represents a frequent clinical challenge in gynecologic practice. While many ovarian cysts remain asymptomatic and regress spontaneously, others cause pelvic pain or abdominal discomfort. In addition, larger cysts may increase the risk of ovarian torsion, prompting interventional treatment. The current standard of care for symptomatic benign ovarian cysts is laparoscopic cystectomy [1]. Compared with open surgery, minimally invasive approaches reduce postoperative pain, hospital stay, and recovery time [2–4]. Nevertheless, laparoscopic surgery remains associated with relevant perioperative risks, with reported intraoperative complication rates of approximately 5.6% and postoperative complications occurring in around 6.5% of cases [5–7]. General anesthesia and abdominal access are required, which may increase perioperative risk, particularly in elderly or multimorbid patients. In premenopausal women, laparoscopic cystectomy may also compromise ovarian reserve due to inadvertent removal of healthy ovarian tissue [8]. Medical treatment with oral contraceptives has not demonstrated superior efficacy compared with expectant management for functional ovarian cysts [9]. Consequently, alternative and less invasive interventional strategies have been explored.

Transvaginal sonography-guided ovarian cyst aspiration represents a minimally invasive approach that avoids abdominal surgery and general anesthesia. The procedure can be performed under local anesthesia, does not require pneumoperitoneum, and eliminates the risk of abdominal access-related complications. Several studies have demonstrated that sonography-guided cyst aspiration is technically feasible and associated with low rates of bleeding, infection, or injury to adjacent organs [10–14]. However, concerns persist regarding oncologic safety. Cytological examination of aspirated cyst fluid has limited sensitivity and cannot reliably exclude malignancy [15, 16]. Therefore, accurate pre-interventional sonographic assessment is essential. The International Ovarian Tumor Analysis (IOTA) group has developed validated sonography-based models that significantly improve discrimination between benign and malignant adnexal masses and support patient selection for conservative management strategies [17, 18]. Another debated aspect of ovarian cyst aspiration is recurrence, with reported rates ranging from less than 10% to more than 70%, depending on cyst type, follow-up duration, and adjunctive therapies [10–14, 19–23]. Recurrence must be balanced against the invasiveness of laparoscopic surgery, particularly in patients with increased surgical risk, where repeat aspiration may represent a reasonable management option. Importantly, intraoperative cyst rupture during laparoscopic cystectomy is not uncommon, even in cases of suspected malignancy, indicating that the risk of cyst rupture is not exclusive to aspiration-based approaches but inherent to ovarian cyst management in general; accordingly, training tools aimed at reducing cyst rupture are gaining popularity [24–27]. Consequently, both surgical and non-surgical strategies require careful patient selection and risk stratification.

Beyond clinical outcomes, health care systems face increasing pressure to reduce costs and resource consumption. While minimally invasive gynecologic surgery has improved patient outcomes compared with open procedures, it is associated with substantial health care expenditures and material use [21, 28–30]. In addition, the health care sector contributes significantly to global greenhouse gas emissions, accounting for approximately 5.2% in Germany and up to 7.6% in the United States [31, 32]. Laparoscopic and robotic procedures are associated with considerable carbon emissions, driven primarily by energy consumption, surgical waste, and anesthesia-related factors [33–37]. Sonography-guided procedures require fewer disposable materials, less energy consumption, and no general anesthesia, suggesting potential economic and environmental advantages [21]. However, before such secondary considerations can be meaningfully addressed, it is essential to establish that transvaginal sonography-guided ovarian cyst aspiration is safe, feasible, and clinically reliable. Therefore, the primary objective of this study was to evaluate the safety and feasibility of transvaginal sonography-guided ovarian cyst aspiration in patients with benign ovarian cysts selected according to IOTA Simple Rules [18]. Secondary objectives were to assess short-term recurrence rates and to contextualize potential economic and environmental implications based on available literature.

## Methods

This retrospective cohort study was conducted at the Hospital of Ehingen, Germany, including female patients aged ≥ 18 years diagnosed sonographically with benign ovarian cysts who underwent transvaginal cyst aspiration between January 2024 and October 2025. Cysts were evaluated using the IOTA Simple Rules by experienced gynecologic sonographers. Given the retrospective design and the use of fully anonymized data, formal ethics approval was not required in accordance with §15 of the Medical Association of Baden-Wuerttemberg’s professional code. All patients had provided consent for the use and processing of their clinical data as part of routine care, so no additional study-specific informed consent was necessary.

All patients underwent transvaginal sonography-guided cyst aspiration under local anesthesia. Conversions to general anesthesia or procedural failures were documented, with patients retained in the analysis. Recorded variables included patient age, menopausal status (defined by age, with women aged 50 years or younger classified as premenopausal), cyst morphology according to IOTA Simple Rules classification [18], cyst location, aspirated fluid volume, and cytological results. No additional risk stratification (e.g., CA-125 or ADNEX score) was performed in postmenopausal patients, apart from IOTA classification. Adverse events such as bleeding or infection were documented. The primary endpoints were procedural feasibility and safety, defined as successful completion of the procedure and absence of major intra- or post-interventional complications. Secondary endpoints included short-term cyst recurrence and exploratory analyses of health care costs and environmental impact based on published literature. Recurrence was identified through self-initiated re-presentation at the hospital, as no standardized follow-up visits or imaging were scheduled. A minimum follow-up time of 6 weeks was defined.

### Cost effectiveness assessment

To assess cost effectiveness, two targeted PubMed (https://pubmed.ncbi.nlm.nih.gov/) searches were conducted:

- “Laparoscopy” AND “Cost” AND “Gynecology”
- “Sonography” AND “Cost” AND “Cyst Aspiration” AND “Gynecology”

Studies published between 2020 and 2025 were included. All reported costs were converted to US dollars (USD) using the European Central Bank currency converter (January 2026: https://data.ecb.europa.eu/currency-converter).

### Carbon footprint assessment

To assess environmental relevance, two targeted PubMed (https://pubmed.ncbi.nlm.nih.gov/) searches were conducted:

- “Laparoscopy” AND “carbon footprint”
- “Sonography” AND “cyst aspiration” AND “carbon footprint”

Eligible studies were analyzed for methodological comparability. Descriptive statistics were performed using Microsoft Excel 2024.

## Results

### Patient characteristics and procedural outcomes

A total of 22 women underwent transvaginal sonography-guided ovarian cyst aspiration between January 2024 and October 2025. The median age was 58 years (range 31–87). Of the study population, 63.6% (*n* = 14) were postmenopausal (> 50 years), whereas 36.4% (n = 8) were premenopausal (≤ 50 years). The median cyst diameter was 5.9 cm (range 2.8–10.0), and the median aspirated volume was 55 mL (range 9–600). According to IOTA classification, most cysts were categorized as B1 (77.3%), with no cases in category B2, one case (4.5%) in B3, and four cases (18.2%) in B4. Ten cysts (45.5%) were located on the right ovary, nine (40.9%) on the left, and in three cases (13.6%) laterality was not specified. Seventeen cysts (77.3%) were located in the Douglas space, four (18.2%) in the retrovesical space, and one (4.5%) at the vaginal stump. The procedure was completed successfully in all but one case, which was terminated due to limited access. Local anesthesia was sufficient in 95% of patients, with conversion to general anesthesia required in one case (4.5%). One cyst recurrence (4.5%) occurred at eight weeks, within a follow-up period of 6 to 47 weeks. No major intra- or post-interventional complications occurred, and there were no cases of bleeding, infection, or injury to adjacent organs (Table 1).

**Table 1:** Transvaginal sonography-guided ovarian cyst aspiration: characteristics and procedure results

Patients: *n* = 22	
Age (in years)	median 58 (range: 31–87)
Menopausal Status	
premenopausal (≤ 50 years)	36.4% (*n* = 8)
postmenopausal (> 50 years)	63.6% (*n* = 14)
Cyst diameter (in cm)	median 5.9 (range: 2.8–10.0)
Fluid (in mL)	median 55 (range: 9–600)
Assessment according to IOTA simple rules	
B1	77.3% (*n* = 17)
B2	0% (*n* = 0)
B3	4.5% (*n* = 1)
B4	18.2% (*n* = 4)
Pelvic location	
right	45.5% (*n* = 10)
left	40.9% (*n* = 9)
Laterality not defined	13.6% (*n* = 3)
Detailed localisation	
Douglas space	77.3% (*n* = 17)
retrovesical space	18.2% (*n* = 4)
vaginal stump	4.5% (*n* = 1)
Complications	
Recurrence of ovarian cyst	4.5% (*n* = 1)
Puncture in anesthesia	4.5% (*n* = 1)
Cessation of puncture due to limited access	4.5% (*n* = 1)

Health care costs of laparoscopic procedures.

The systematic literature review assessing health care costs of laparoscopic and robotic procedures in gynecology identified 29 publications in PubMed, including clinical studies, clinical trials, and randomized controlled trials. After screening titles, abstracts, and full texts, 25 studies were excluded due to insufficient, incomplete, or non-comparable cost reporting. Ultimately, four studies were included in the final analysis.

The included studies comprised between 13 and 159 patients per study and evaluated a range of gynecologic procedures, including surgical staging for early-stage endometrial cancer in obese patients, hysterectomy for gynecologic malignancy, laparoscopically assisted vaginal hysterectomy, and laparoscopic ovarian endometrioma cystectomy. Both laparoscopic and robotic approaches were represented, with two studies directly comparing these access modalities [28, 29]. The studies were conducted in Canada, Great Britain, and South Africa and collectively included 463 patients.

Across all included studies, robotic surgery was consistently associated with higher costs compared with laparoscopic approaches. In obese patients undergoing surgical staging for early-stage endometrial cancer, Kosa et al. reported mean surgical costs of USD 3979.01 for laparoscopic access compared with USD 5203.78 for robotic access [29]. Similarly, McCarthy et al. documented mean surgical costs of USD 5018.06 for laparoscopic hysterectomy and USD 6382.23 for robotic hysterectomy performed for gynecologic malignancy [28]. In contrast, Chrysostomou et al. reported substantially lower surgical costs for laparoscopically assisted vaginal hysterectomy, with a mean cost of USD 740.18 [30].

When overall health care costs were considered, including perioperative and associated expenditures, a comparable pattern emerged. Total costs for laparoscopic and robotic access in early-stage endometrial cancer amounted to USD 6059.03 and USD 6483.86, respectively [29]. For hysterectomy in the context of gynecologic malignancy, overall costs were USD 6159.56 for laparoscopic surgery and USD 7326.55 for robotic surgery [28]. Lower overall costs were reported for laparoscopically assisted vaginal hysterectomy (USD 1324.35) and laparoscopic ovarian endometrioma cystectomy (USD 2475.08) [21, 30]. Across all included surgical procedures, the mean surgical cost was USD 4264.65, and the mean overall health care cost was USD 4971.41 (Table 2).

**Table 2:** Assessment of health care costs for gynecologic laparoscopic, robotic, and ultrasound-guided procedures: systematic PubMed search results

Study	Kosa et al. 2021 [29]	Kosa et al. 2021 [29]	Chrysostomou et al. 2021 [30]	McCarthy et al. 2023 [28]	McCarthy et al. 2023 [28]	Garcia-Tejedor et al. 2025 [21]	Garcia-Tejedor et al. 2025 [21]
Procedure	Early endometrial cancer in obese patients (Laparoscopic)	Early endometrial cancer in obese patients (Robotic)	Laparoscopically-assisted vaginal hysterectomy	Hysterectomy for gynaecological malignancy (Laparoscopic)	Hysterectomy for gynaecological malignancy (Robotic)	Laparoscopic ovarian endometrioma cystectomy	Ultrasound-guided aspiration with ethanol sclerotherapy of ovarian endometriomas
Number of Patients (n)	13	68	76	73	159	74	84
Country of Trial	Canada	Canada	South Africa	Great Britain	Great Britain	Spain	Spain
Surgery Cost (USD)	$ 3979.01	$ 5203.78	$ 740.18	$ 5018.06	$ 6382.23		
Overall Cost (USD)	$ 6059.03	$ 6483.86	$ 1324.35	$ 6159.56	$ 7326.55	$ 2475.08	$ 548.98

The systematic review assessing health care costs of sonography-guided cyst aspiration identified four publications. Among these, one prospective, randomized multicenter trial reported detailed cost outcomes for transvaginal sonography-guided aspiration with ethanol sclerotherapy of ovarian endometriomas. In this study, 84 patients underwent sonography-guided sclerotherapy, with a reported mean overall health care cost of USD 548.98 per procedure [21].

Although the included studies addressed different gynecologic procedures and were conducted in heterogeneous health care systems, sonography-guided interventions were associated with markedly lower reported health care costs compared with laparoscopic and robotic surgery. However, due to the limited number of eligible studies reporting standardized cost data and the lack of direct comparisons within the same clinical setting, quantitative cross-modality comparisons remain restricted.

Carbon footprint assessment of laparoscopic procedures.

The systematic review assessing the carbon footprint of laparoscopic and robotic procedures retrieved 23 publications. Nineteen studies were excluded due to insufficient data on emissions per procedure, leaving four studies eligible for inclusion. These included between 15 and 128 patients each and covered both general and gynecologic surgical procedures, such as staging for endometrial cancer, hysterectomy, and cholecystectomy. Two studies directly compared laparoscopic and robotic approaches. Among the included studies, Woods et al. (2015) reported carbon emissions of 40.3 kg CO^2^e per robotic and 29.2 kg CO^2^e per laparoscopic staging procedure for endometrial cancer, with waste contributing 14.3 kg and 11.2 kg CO^2^e, respectively [34]. Comes et al. (2024) documented a mean total footprint of 56.5 kg CO^2^e per laparoscopic cholecystectomy, composed of 6.1 kg from energy use, 22.7 kg from waste, and 27.7 kg from others like anesthesia-related emissions [35]. In Ramani et al. (2023), the reported waste-related emissions were 10.7 kg CO^2^e for laparoscopic and 12.01 kg CO^2^e for robotic hysterectomy [36]. Gangakhedkar et al. (2025) described 4.04 kg CO^2^e waste emissions per laparoscopic cholecystectomy [37]. Across all studies, waste-related emissions were reported in every analysis and served as a comparable parameter. The mean CO^2^ emission from surgical waste was 12.49 kg per procedure. When energy consumption was measured (in two studies, n = 115), the mean CO^2^ emission from energy use was 16.7 kg per procedure. The overall mean carbon footprint per laparoscopic procedure was 42 kg CO^2^e, with laparoscopic approaches averaging 12.16 kg CO^2^e and robotic procedures 13.16 kg CO^2^e from waste alone (Table 3).

**Table 3:** Assessment of carbon footprint impact: systematic PubMed search results

Study	Woods et al. 2015 [34]	Woods et al. 2015 [34]	Ramani et al. 2023 [36]	Ramani et al. 2023 [36]	Comes et al. 2024 [35]	Gangakhedkar et al. 2025 [37]
Procedure	Staging Procedure for Endometrial Cancer	Staging Procedure for Endometrial Cancer	Hysterectomy	Hysterectomy	Cholecystectomy	Cholecystectomy
Endoscopic Access	Robotic	Laparoscopy	Laparoscopy	Robotic	Laparoscopy	Laparoscopy
Number of Patients	50	50	34	56	15	128
Total Carbon footprint (kg CO^2^e/patient)	40.3	29.2			56.5	
Energy (kg in CO^2^e/patient	26	18			6.1	
Waste (kg CO^2^e/patient	14.3	11.2	10.7	12.01	22.7	4.04
Others f.e. anaesthesia (kg CO^2^e/patient)					27.7	

No publications were identified in PubMed that addressed the carbon footprint of sonographically guided cyst aspiration. Therefore, direct environmental comparison between laparoscopic and sonographic approaches could not be performed.

## Discussion

This retrospective cohort study demonstrates that transvaginal sonography-guided ovarian cyst aspiration is a safe and technically feasible procedure for the management of carefully selected benign ovarian cysts. In this cohort, the intervention was successfully completed in nearly all patients, predominantly under local anesthesia, and was not associated with major intra- or post-interventional complications. These findings support previous reports describing low rates of bleeding, infection, or injury to adjacent organs following sonography-guided cyst aspiration [10–14, 19–23]. The high procedural feasibility observed in this study is clinically relevant, particularly for patients in whom general anesthesia or abdominal surgery carries increased risk. In contrast to laparoscopic cystectomy, transvaginal aspiration avoids pneumoperitoneum, abdominal trocar placement, and prolonged anesthesia time. Laparoscopic gynecologic surgery, although widely accepted as the standard of care, is still associated with intraoperative complication rates of approximately 5–6% and postoperative complication rates exceeding 6% [5–7]. These risks must be considered when managing older patients, those with significant comorbidities, or patients who explicitly prefer a less invasive approach.

From an oncologic safety perspective, accurate preinterventional selection of patients remains paramount. Cytological examination of aspirated cyst fluid has limited sensitivity and specificity and cannot reliably exclude malignancy [15, 16]. Consequently, the decision to perform cyst aspiration should never rely on cytology alone. In the present study, all cysts were classified as benign according to IOTA Simple Rules, which have been extensively validated and shown to significantly improve discrimination between benign and malignant adnexal masses [17, 18]. Strict adherence to such sonographic criteria is essential to minimize oncologic risk when considering conservative interventional approaches. Recurrence of ovarian cysts following transvaginal sonography-guided aspiration remains highly variable, with reported rates ranging from under 10% to over 70%, depending on cyst type, follow-up duration, and use of adjunctive measures such as sclerotherapy (Table 4) [10–14, 19–23]. In our cohort of 22 women, the majority were postmenopausal (63.6%), a higher proportion than most published studies. Only one recurrence (4.5%) was observed, detected solely through patient self-presentation, as no structured follow-up was implemented. This likely underestimates true recurrence and highlights the impact of follow-up intensity on reported rates. Compared with the summary data from Table 4, our study reflects a similar low recurrence rate to other non-sclerotherapy studies (mean recurrence 48.3%), while sclerotherapy studies report lower recurrence (mean recurrence 16.4%) but typically include more structured follow-up. Importantly, recurrence does not necessarily imply treatment failure, as repeat aspiration can often be performed with minimal additional risk and burden to the patient. When comparing aspiration with laparoscopic cystectomy, it should also be acknowledged that intraoperative cyst rupture is not uncommon during surgical removal [24–26]. Therefore, the potential risk of cyst rupture is not exclusive to aspiration-based approaches but is inherent to ovarian cyst management in general. This further underscores the importance of careful patient selection and shared decision-making rather than reliance on a single management strategy.

**Table 4:** Comparative analysis of studies on transvaginal sonography-guided ovarian cyst treatment

Study	Number of patients	Premenopausal	Postmenopausal	Mean Follow-up (in months)	Number of Follow-ups	Sclerotherapy	Major Morbidity	Recurrence Rate
Present study	22	36.4% (*n* = 8)	63.6% (*n* = 14)			no	0	4.5% (*n* = 1)
Petrovic et al. 2002 [13]	72	76.4% (*n* = 55)	23.6% (*n* = 17)		2	no	1.4% (*n* = 1)	44.4% (*n* = 32)
Dilbaz et al. 2003 [12]	35	88.6% (*n* = 31)	11.4% (*n* = 4)	12	1	no	0	8.6% (*n* = 3)
Duke et al. 2006 [10]	24	58.3% (*n* = 14)	41.7% (*n* = 10)	39.5		no	0	75% (*n* = 18)
Koutlaki et al. 2011 [14]	121	86% (*n* = 104)	9.9% (*n* = 12)	6	1	no	0	33.1% (*n* = 40)
Nikolaou et al. 2014 [11]	46	73.9% (*n* = 34)	26.1% (*n* = 12)	6	3	no	0	39.1% (*n* = 18)
Wu and Xu et al. 2015 [22]	34			12	3	yes	0	35.3% (*n* = 12)
Castellarnau et al. 2016 [19]	66	42.4% (*n* = 28)	54.5% (*n* = 36)	26.5	0	no	0	72.7% (*n* = 48)
Castellarnau et al. 2016 [19]	75	42.7% (*n* = 32)	54.7% (*n* = 41)	20.3	0	yes	0	22.7% (*n* = 17)
Fei et al. 2018 [23]	110	100% (*n* = 110)	0% (*n* = 0)	6	3	yes	0	7.3% (*n* = 8)
Diaz de la Noval et al. 2020 [20]	156	42.3% (*n* = 66)	57.7% (*n* = 90)	18,5	2	no	1.9% (*n* = 3)	65.4% (*n* = 102)
Garcia-Tejedor et al. 2025 [21]	25	100% (*n* = 25)	0% (*n* = 0)	17	4	yes	0	12% (*n* = 3)
Summary: sclerotherapy = no	542	62.7% (*n* = 340)	36.0% (*n* = 195)	Mean: 13.8	Mean: 1.8	no	0.7% (*n* = 4)	48.3% (*n* = 262)
Summary: sclerotherapy = yes	244	68.4% (*n* = 167)	16.8% (*n* = 41)	Mean: 13.8	Mean: 3.3	yes	0% (*n* = 0)	16.4% (*n* = 40)

From an economic perspective, the literature review performed in this study demonstrates marked differences in health care costs between surgical and sonography-guided approaches. Minimally invasive gynecologic procedures, particularly robotic surgery, are consistently associated with higher surgical and overall health care costs compared with laparoscopic access [28, 29]. In contrast, sonography-guided cyst aspiration and sclerotherapy have been reported to incur substantially lower overall costs [21]. Although the included studies evaluated different gynecologic procedures across various health care systems, the magnitude of the observed cost differences suggests that sonography-guided approaches may offer significant economic advantages, especially when performed on an outpatient basis. Nevertheless, it must be acknowledged that higher recurrence rates could offset these advantages if multiple repeat interventions are required, an aspect that should be addressed in future cost-effectiveness analyses. The environmental dimension of surgical care is an emerging field of research with increasing relevance. Available data indicate that laparoscopic and robotic procedures are associated with considerable carbon footprints, driven primarily by energy consumption, disposable instruments, and anesthesia-related emissions [33–37]. The mean reported carbon footprint of approximately 42 kg CO^2^e per laparoscopic procedure highlights the environmental impact of modern surgical care. No data are currently available regarding the carbon footprint of transvaginal sonography-guided ovarian cyst aspiration. However, given its minimal equipment requirements, avoidance of general anesthesia, and limited use of disposable materials, it is plausible that its environmental impact is substantially lower. These considerations remain exploratory and hypothesis-generating but point toward an important area for future research.

Taken together, the findings of this study support transvaginal sonography-guided ovarian cyst aspiration as a safe and feasible management option for selected patients with benign ovarian cysts. While it cannot replace surgical management in all cases, it may represent a valuable complementary approach, particularly in patients with increased surgical risk or a preference for minimally invasive treatment.

### Limitations

This study has several limitations that must be considered when interpreting the results. First, the retrospective design and small sample size limit statistical power and generalizability. Second, follow-up was not standardized, and recurrence was identified only when patients re-presented to the hospital, which may have led to underestimation of true recurrence rates. Especially since the minimum observation period was defined as 6 weeks. Third, apart from the application of the IOTA Simple Rules, no further risk stratification or blood serum analysis was performed in either premenopausal or postmenopausal patients. Fourth, the cost analysis was based on a systematic review of heterogeneous studies conducted in different countries and across different gynecologic procedures. Variations in national health care systems, reimbursement structures, labor costs, and perioperative pathways limit direct comparability. Fifth, the environmental assessment relied exclusively on published data for laparoscopic and robotic procedures, many of which were not exclusively gynecologic. Key parameters such as procedure duration, instrument reuse, and anesthesia type were inconsistently reported, precluding precise comparison. No direct life-cycle assessment data exist for sonographically guided ovarian cyst aspiration, and any assumptions regarding its carbon footprint must therefore be interpreted cautiously. Finally, patient-reported outcomes such as pain, satisfaction, and quality of life were not assessed in this study but represent important endpoints for future research.

## Conclusion

Transvaginal sonography-guided ovarian cyst aspiration demonstrated a favorable safety profile, high procedural feasibility, and low short-term recurrence in patients with ovarian cysts classified as benign according to IOTA Simple Rules. The procedure avoids general anesthesia and abdominal surgery, thereby reducing patient morbidity. Based on available literature, it may also offer economic advantages and reduced resource consumption compared with minimally invasive surgical approaches. However, given the limited sample size, this technique should be considered a complementary or alternative option in selected patients rather than a first-line treatment. Larger prospective studies are required to confirm these findings.

## Data Availability

No datasets were generated or analysed during the current study.
